# The Complexities of Sepsis-Induced Cardiomyopathy: A Clinical Case and Review of Inflammatory Pathways and Potential Therapeutic Targets

**DOI:** 10.7759/cureus.75173

**Published:** 2024-12-05

**Authors:** Pawel Borkowski, Michal Borkowski, Natalia Borkowska, Vishakha Modak, Natalia Nazarenko, Shaunak Mangeshkar, Anita Osabutey, Maisha Maliha, Ishmum Chowdhury, Ashot Batikyan, Bisrat Adal, Vikyath Satish

**Affiliations:** 1 Internal Medicine, Albert Einstein College of Medicine, Jacobi Medical Center, New York, USA; 2 Medicine, Private Practice, Wroclaw, POL; 3 Pediatrics, Samodzielny Publiczny Zaklad Opieki Zdrowotnej - Krotoszyn, Krotoszyn, POL; 4 Internal Medicine, Jacobi Medical Center, New York, USA; 5 Internal Medicine, Albert Einstein College of Medicine, Jacobi Medical Center/North Central Bronx Hospital, New York, USA; 6 Internal Medicine, Kempegowda Institute of Medical Sciences, Bangalore, IND

**Keywords:** inflammatory response, left ventricle ejection fraction, sepsis-induced cardiomyopathy, septic cardiomyopathy, severe sepsis

## Abstract

Sepsis-induced cardiomyopathy (SICM) is a life-threatening complication of sepsis characterized by myocardial dysfunction. SICM significantly increases mortality rates in sepsis. Despite its clinical relevance, SICM lacks a unified definition and standardized diagnostic criteria, complicating early identification and treatment. The pathophysiology of SICM is complex and involves a combination of inflammatory mediators, oxidative stress, mitochondrial dysfunction, and dysregulated autonomic responses. Cardiac biomarkers such as B-type natriuretic peptide, N-terminal pro-B-type natriuretic peptide, and troponins provide valuable prognostic insights but lack specificity for SICM diagnosis. This article presents the case of a 69-year-old woman who experienced rapid left ventricular dysfunction, initially misattributed to acute coronary syndrome but ultimately diagnosed as SICM. Her condition improved significantly after several days of supportive care, with full recovery of left ventricular function, highlighting the reversible nature of SICM. This article discusses the underlying pathophysiological mechanisms behind SICM, the utility of cardiac biomarkers, and potential therapies addressing specific molecular pathways. Current management of SICM primarily emphasizes supportive care and hemodynamic stabilization. Further research is essential to develop more precise diagnostic tools and effective treatments for this complex and underrecognized condition. Addressing these gaps could significantly reduce treatment delays and improve patient outcomes.

## Introduction

Sepsis is a severe, life-threatening condition defined by organ dysfunction resulting from a dysregulated immune response to infection [1]. Globally, sepsis remains a significant public health burden, with an estimated 48.9 million cases and 11 million related deaths reported in 2017 [2]. In the United States, sepsis disproportionately affects older adults, with a mortality rate of 330.9 per 100,000 individuals aged 65 and older in 2021 [3]. Notable racial disparities in sepsis outcomes persist, with Black patients facing higher mortality rates than non-Hispanic Whites, reflecting broader healthcare inequities [4,5]. Cardiovascular complications of sepsis have been documented for over five decades, and Parker et al. were one of the first researchers to describe sepsis-induced cardiomyopathy (SICM) in 1984 [6]. These cardiovascular abnormalities significantly worsen prognosis, as SICM in the context of septic shock is associated with mortality rates as high as 70% to 90% [7]. Despite its clinical relevance, a unified definition of SICM remains unclear, and its prevalence among patients with sepsis is estimated to be 10% to 70%, depending on the diagnostic criteria used [8]. This variability arises from evolving sepsis definitions, differences in imaging modalities, inconsistent echocardiographic criteria, methodological discrepancies, and challenges in clinical recognition. These factors underscore the urgent need for consensus guidelines to standardize the diagnosis of SICM.

Cardiomyopathy often manifests with symptoms such as dyspnea, fatigue, reduced exercise capacity, orthopnea, paroxysmal nocturnal dyspnea, palpitations, and syncope [9]. Hemodynamic compromise due to reduced cardiac output is a common feature, aligning SICM with other forms of cardiomyopathy. However, SICM is distinct in its rapid onset and characteristic features - global ventricular dysfunction, left ventricular dilatation with normal or low filling pressures, and reduced ejection fraction without regional wall motion abnormalities [10,11]. A critical differentiating factor of SICM is its reversibility; most cases demonstrate significant recovery of cardiac function by the 10th day after symptom onset, despite its potential to acutely elevate mortality risk [6]. Understanding the unique pathophysiology and clinical course of SICM is crucial for optimizing sepsis management and improving patient outcomes.

## Case presentation

**Figure 1:**
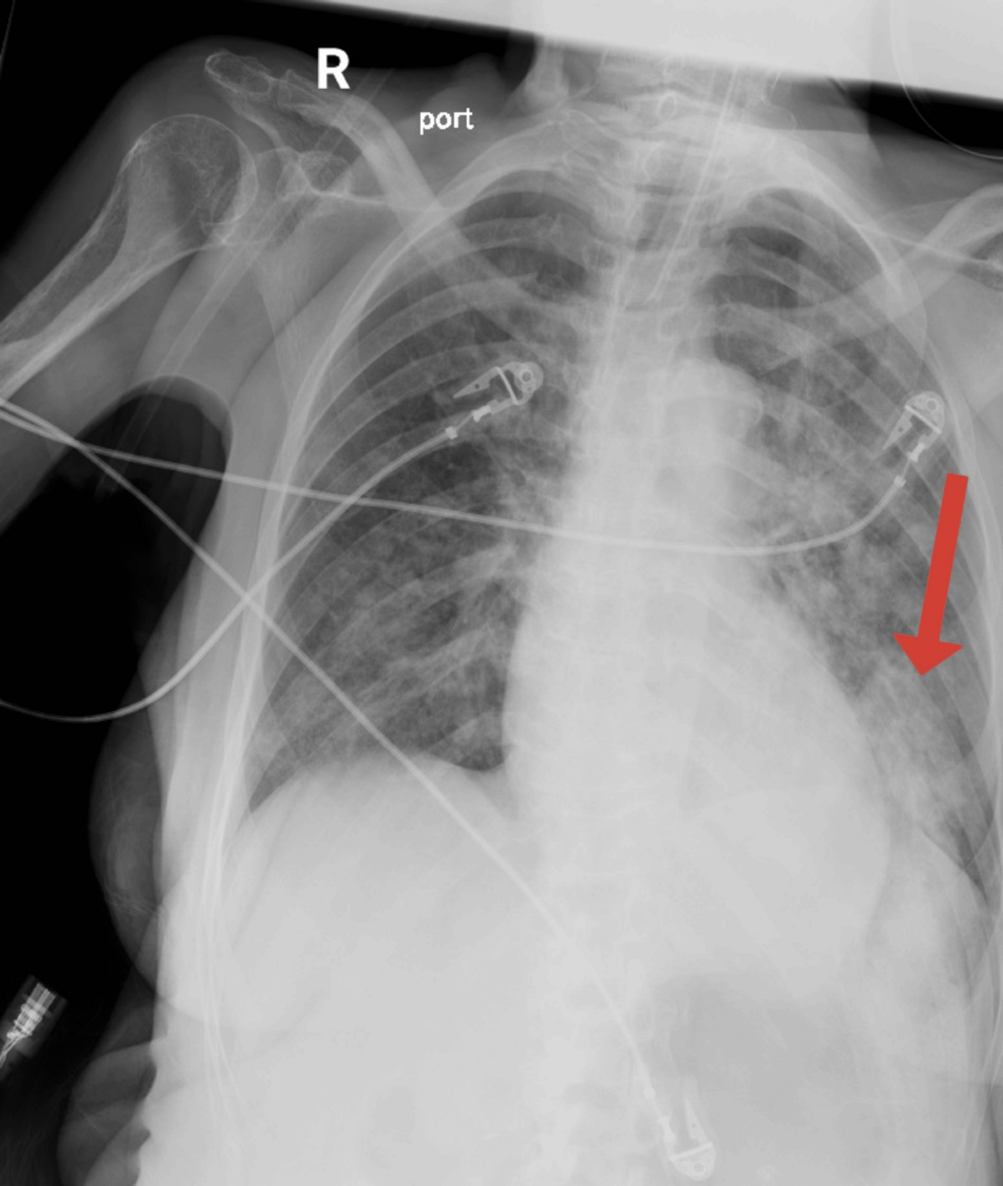
Bilateral patchy opacities are noted, more confluent in the lower lobes, with greater involvement on the left compared to the right (red arrow), consistent with pneumonia. The endotracheal tube is in a satisfactory position. A right internal jugular central venous catheter terminates appropriately at the superior vena cava/right atrial junction.

**Figure 2:**
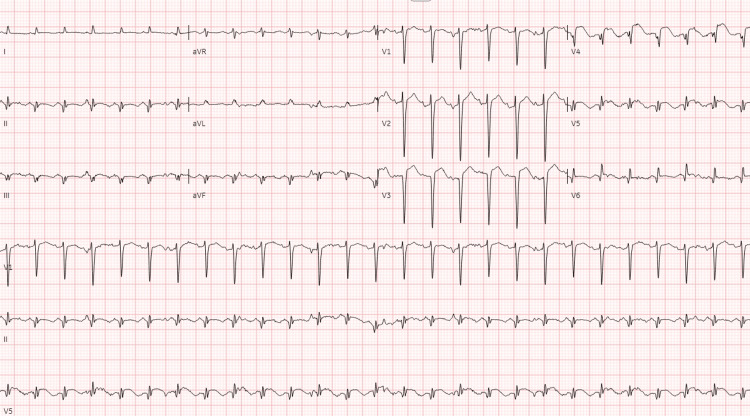
Multifocal atrial tachycardia with a heart rate of 161 bpm, left axis deviation, Q waves in the anterolateral and inferior leads, and ST-segment elevation in the anterolateral leads.

A 69-year-old woman with a medical history significant for hypertension, hyperlipidemia, and type 2 diabetes presented to the emergency department with respiratory distress. She had been receiving intravenous antibiotics for sacral osteomyelitis at a Long-Term Acute Care Hospital prior to her presentation. Over the past few days, she experienced progressive weakness, decreased oral intake, and the development of dyspnea and hypoxia, leading to her transfer to the hospital. Upon arrival, the patient's vital signs were as follows: temperature 38.8°C, heart rate 130 bpm, blood pressure 100/50 mmHg, respiratory rate 40 breaths/min, and oxygen saturation 80% on room air. Physical examination revealed signs of acute respiratory distress, bilateral basal crackles, tachycardia, cool extremities, and delayed capillary refill. Despite initial support with a non-rebreather mask and later bilevel positive airway pressure (BiPAP), the patient's oxygenation did not improve, necessitating intubation. Laboratory results showed leukocytosis, lymphopenia, elevated procalcitonin, transaminitis, elevated lactic acid, and elevated troponin T (Table 1). Urine Streptococcus pneumoniae antigen and COVID-19 rapid antigen test were both positive. Chest x-ray indicated multifocal pneumonia (Figure 1). An electrocardiogram revealed multifocal atrial tachycardia with a rate of 161 bpm (attributed to hypoxia), left axis deviation, Q waves in the anterolateral and inferior leads, and ST-segment elevation in the anterolateral leads (Figure 2). Bedside ultrasound demonstrated severely decreased left ventricular function (ejection fraction was estimated to be 60% one year prior). As her condition deteriorated, her blood pressure dropped to 88/50 mmHg. Broad-spectrum antibiotics, norepinephrine infusion, and stress-dose steroids were initiated, and the patient was admitted to the medical ICU for further management.

**Table 1:** Laboratory results.

Laboratory Test	Actual Result	Normal Range
White blood cells (WBC)	12.57 K/uL	3.5 – 11 K/uL
Neutrophils (absolute)	10.71 K/uL	1.7 – 9 K/uL
Lymphocytes (absolute)	1.02 K/uL	1.2 – 3.5 K/uL
Hemoglobin (HGB)	9.8 g/dL	12 – 16 g/dL
Platelets (PLT)	307 K/uL	150 – 440 K/uL
Creatinine (Cr)	0.8 mg/dL	0.5 – 1.5 mg/dL
Blood urea nitrogen (BUN)	18 mg/dL	5 – 26 mg/dL
Sodium (Na)	137 mEq/L	135 – 145 mEq/L
Potassium (K)	4.2 mEq/L	3.5 – 5 mEq/L
Alanine aminotransferase (ALT)	42 U/L	10 – 40 U/L
Aspartate transferase (AST)	94 U/L	10 – 40 U/L
Anion gap (AG)	21.5 mEq/L	≤ 13.9 mEq/L
Bicarbonate	22.5 mEq/L	24 – 30 mEq/L
pH	7.39	7.32 – 7.43
Partial pressure of CO2 (PCO2)	42 mmHg	38 – 41 mmHg
Lactic acid	8.5 mmol/L	0.6 – 1.4 mmol/L
Troponin T (High sensitivity)	909 ng/L	0 – 0.14 ng/L
Creatine kinase (CK)	335 U/L	5 – 150 U/L
C-reactive protein (CRP)	283.3 mg/L	0 – 5 mg/L
Procalcitonin (PCT)	4.09 ng/mL	0.02 – 0.08 ng/mL

Cardiac output, calculated from central venous oxygen saturation (ScvO2) using the Fick method, was estimated at 2.2 L/min, with a cardiac index of 1.6 L/min/m². Cardiology was consulted due to concerning EKG changes and elevated troponin levels. Initially, cardiology suspected a resolved myocardial infarction, with no recommendation for left heart catheterization due to hemodynamic instability. Over the next eight hours, the patient’s troponin T level rose from 909 ng/L to 1210 ng/L, remaining stable. However, subsequent measurements showed a significant increase, with a level of 2191 ng/L. At this point, the patient was started on a heparin drip and received dual antiplatelet therapy. Due to the patient’s unstable condition, diagnostic and/or therapeutic left heart catheterization was deferred. A transthoracic echocardiogram revealed diffuse left ventricular hypokinesis with an ejection fraction of 15%, left ventricle end-diastolic diameter (LVEDD) of 3.6 cm, and no signs of elevated left ventricular end-diastolic pressure or regional wall motion abnormalities (Video 1). A repeat transthoracic echocardiogram seven days later showed significant improvement in left ventricular function, with an ejection fraction of 55%, and again LVEDD of 3.6 cm and no signs of elevated left ventricular end-diastolic pressure or regional wall motion abnormalities (Video 2).

**Video 1:** Echocardiogram showing severe diffuse left ventricular hypokinesis with a markedly reduced left ventricular ejection fraction of 15%. The ejection fraction was evaluated by the biplane method of disks.

**Video 2:** Echocardiogram showing a normal left ventricular ejection fraction of 55%. The ejection fraction was evaluated by the biplane method of disks.

The patient spent nearly two months in the hospital, where she was managed for mixed septic and cardiogenic shock, which ultimately resolved. Her hospital course was complicated by urinary retention, acute tubular necrosis, deconditioning, and a urinary tract infection. All complications were resolved, and she was discharged home. The transient cardiac dysfunction was attributed to SICM rather than acute coronary syndrome. Supporting evidence for this diagnosis includes the rapid recovery of left ventricular function, absence of regional wall motion abnormalities, and normal left ventricular end-diastolic filling pressures.

## Discussion

Initially, it was thought that SICM was a result of ischemic heart disease. Research has shown that coronary perfusion is paradoxically increased in patients with septic shock, likely due to the effects of vasodilatory molecules released during sepsis [12]. This discovery has shifted the focus toward the biochemical mediators in the pathogenesis of SICM. Numerous myocardial depressant factors have been implicated, including cytokines, prostanoids, components of the complement cascade, nitric oxide, oxidative stress, mitochondrial dysfunction, and pathogen-associated molecular patterns (PAMPs) [7,13]. Beyond biochemical factors, specific clinical and laboratory findings are associated with an elevated risk and/or worse prognosis of SICM. These include male sex, younger age, elevated NT-proBNP (N-terminal Pro-B-type natriuretic peptide) levels, increased lactate concentrations, positive blood cultures, hypoalbuminemia, and a history of heart failure [7,14,15]. Together, these risk factors and mediators highlight the heterogeneous nature of SICM.

Proinflammatory cytokines, including tumor necrosis factor (TNF), interleukin-1 (IL-1), and interleukin-6 (IL-6), are well-recognized mediators of SICM [7]. These cytokines impair cardiac contractility and play an important role in the development of septic myocardial dysfunction, primarily by amplifying the inflammatory response and its downstream consequences, as outlined below [16-18]. Patients with sepsis demonstrate elevated levels of prostanoids, such as thromboxane and prostacyclin, which are known to disrupt coronary endothelial function [13]. Sepsis also triggers robust complement activation, with the C5a component emerging as a significant contributor to SICM [19,20]. C5a binds to receptors on cardiomyocytes, triggering the production of reactive oxygen species (ROS) and increasing the amount of intracellular calcium, which together lead to impaired contractility [21]. Nitric oxide, synthesized by nitric oxide synthase in various cell types including cardiac myocytes, is another key mediator. It induces oxidative stress, reduces the sensitivity of myofibrils to calcium, and downregulates β-adrenergic receptors, collectively contributing to reduced myocardial contractility [22,23]. The massive inflammatory response associated with sepsis is also linked to excessive ROS production, which oxidizes myofibrillar proteins, further exacerbating contractile dysfunction [24]. Given the high mitochondrial density in cardiomyocytes, mitochondrial dysfunction has a profound impact on cardiac function [25]. Structural changes such as swelling, internal vesicle formation, combined with mtDNA damage, and increased mitochondrial permeability disrupt energy production [26,27]. The resultant ATP depletion compromises both systolic contraction and diastolic relaxation of the heart. PAMPs, such as lipopolysaccharide, lipoteichoic acid, bacterial DNA, viral nucleic acids, and fungal mannan, further amplify this process by activating pattern-recognition receptors on host cell surfaces [28]. This interaction activates a proinflammatory cascade described above, intensifying the cardiodepressive effects and contributing to the pathophysiology of SICM.

The two most extensively studied biomarkers associated with SICM are natriuretic peptides and the troponin complex. B-type natriuretic peptide (BNP) and its inactive precursor, NT-proBNP, are produced and released by the ventricular myocardium in response to myocardial wall stress [29,30]. The troponin complex, consisting of troponin C (cTnC), troponin I (cTnI), and troponin T (cTnT), plays a role in muscle contraction [31,32]. Specifically, cTnI and cTnT are biomarkers of cardiac myocyte damage. The mechanisms underlying the elevation of BNP and NT-proBNP in sepsis remain unclear. While these peptides are typically elevated due to myocardial stretch, studies have shown that factors like pulmonary capillary wedge pressures and volume expansion do not correlate with natriuretic peptide levels in sepsis [33]. Other potential contributors to elevated natriuretic peptides include systemic inflammatory response, vasopressor use, and kidney injury, as NT-proBNP is cleared by the kidneys [34]. Growing evidence suggests that BNP and NT-proBNP could serve as valuable predictors of in-hospital mortality in sepsis and septic shock [33,35,36]. The precise mechanisms of myocyte injury in sepsis, particularly in the absence of acute coronary syndrome, leading to elevated troponin levels, remain incompletely understood. Proposed factors include inflammatory mediators, endotoxins, increased myocardial cell membrane permeability, and microvascular dysfunction [37]. Additionally, a mismatch between myocardial oxygen demand and supply may contribute to the observed elevation in troponin levels during sepsis. Elevated troponin level is related to an increased risk of death in sepsis [38]. One study found that NT-proBNP outperformed hs-cTnT in predicting 90-day mortality in patients with septic shock [39]. However, it failed to accurately identify septic cardiomyopathy due to insufficient specificity in this analysis. In conclusion, while elevated cardiac biomarkers are established indicators of prognosis in sepsis, none are specific enough to diagnose SICM, and there are no definitive cut-off values to guide clinical practice.

Given the potential of stopping biochemical pathways involved in SICM, targeted interventions may help address the severe inflammatory response. For example, Zhang et al. found that melatonin could mitigate sepsis-induced myocardial depression by modulating B-cell receptor-associated protein 31 (BAP31), thereby preserving cardiac function in SICM [40]. Furthermore, Kim et al. found that glucose-insulin-potassium (GIK) administration improved hemodynamics in septic shock patients with myocardial depression, without causing additional adverse effects [41]. The authors attributed this benefit to insulin's ability to suppress the secretion of proinflammatory cytokines, such as TNF-α. In addition, Gao et al. found that Schistosoma japonicum-derived cystatin (Sj-Cys) significantly reduced SICM [42]. Furthermore, Bagate et al. demonstrated that low-dose steroid administration in septic patients resulted in increased arterial pressure, decreased heart rate, and improved left ventricular contractility [43]. The studies mentioned above propose several potential therapeutic agents for the prevention and treatment of SICM. These agents appear to target various inflammatory pathways, indicating that inhibiting inflammation may offer a promising approach to managing SICM.

## Conclusions

SICM remains a significant cause of morbidity and mortality in septic shock, with complex and multifactorial pathophysiology. Despite its clinical importance, SICM lacks a unified definition and standardized diagnostic criteria, complicating early identification and management. The rapid onset and reversible nature of SICM make it distinct from other cardiomyopathies, but its association with high mortality underscores the need for more effective treatments. Current biomarkers, including BNP, NT-proBNP, and troponins, provide valuable prognostic information in sepsis, but their inability to specifically diagnose SICM highlights the need for more targeted diagnostic tools. Research suggests that various biochemical pathways, particularly those involving inflammatory mediators, play a central role in the development of SICM. Targeted therapies, including melatonin, GIK, Schistosoma japonicum-derived cystatin, and steroids, show promise in reducing myocardial depression in SICM. Further research is needed to refine diagnostic criteria and develop targeted therapies to effectively manage SICM.
